# Association between time spent on smartphones and digital eye strain: A 1-year prospective observational study among Hong Kong children and adolescents

**DOI:** 10.1007/s11356-023-26258-0

**Published:** 2023-03-29

**Authors:** Geoffrey C. H. Chu, Lily Y. L. Chan, Chi-wai Do, Andy C. Y. Tse, Teris Cheung, Grace P. Y. Szeto, Billy C. L. So, Regina L. T. Lee, Paul H. Lee

**Affiliations:** 1 School of Optometry, The Hong Kong Polytechnic University, Hong Kong, Hong Kong; 2 Research Centre for SHARP Vision (RCSV), The Hong Kong Polytechnic University, Hong Kong, Hong Kong; 3 Department of Health and Physical Education, Education University of Hong Kong, Hong Kong, Hong Kong; 4 School of Nursing, The Hong Kong Polytechnic University, Hong Kong, Hong Kong; 5 School of Medical and Health Sciences, Tung Wah College, Hong Kong, Hong Kong; 6 Department of Rehabilitation Sciences, The Hong Kong Polytechnic University, Hong Kong, Hong Kong; 7 Nethersole School of Nursing, Chinese University of Hong Kong, Hong Kong, Hong Kong; 8 Southampton Clinical Trials Unit, University of Southampton, Southampton General Hospital, Tremona Road, Southampton, SO16 6YD Hampshire UK

**Keywords:** Smartphones, Blurred vision, Digital eye strain, Chinese, Eye fatigue, Longitudinal study, Schoolchildren, Adolescents

## Abstract

Prolonged electronic screen use can cause digital eye strain. It can be difficult to rectify due to increasing smartphone reliance, potentially leading to serious public health problems. To investigate the association between time spent on smartphones and digital eye strain (DES) among Hong Kong Chinese school-aged children. Of a total of 1,508 students (748 males, 49.6%) from 8 to 14 years old (mean age = 10.91 years, SD = 2.01) who provided valid data on DES, the 1,298 (86%) who completed the DES questionnaire at 1-year follow-up were included in the analysis. DES was measured using a 10-item scale, and the sum of the 10 dichotomised scores was used as the DES total score. The most commonly reported symptoms were eye fatigue (n = 804, 53.3%), blurred vision (changing from reading to distance viewing) (n = 586, 38.9%), and irritated or burning eyes (n = 516, 34.2%). The DES total scores at baseline and 1-year follow-up were 2.91 (SD = 2.90) and 3.20 (SD = 3.19), respectively. Linear regression controlling for demographic and socio-economic confounders showed that participants with baseline smartphone usage of 241 + min/d had a significantly higher baseline total DES score than those with baseline smartphone usage of 0–60 min/d (2.44 vs 3.21, *P* < 0.001), and participants with baseline smartphone usage of 181–240 min/d had a significantly higher 1-year follow-up total DES score than those with baseline smartphone usage of 0–60 min/d (2.80 vs 3.50, *P* = 0.003).

**Supplementary Information:**

The online version contains supplementary material available at 10.1007/s11356-023-26258-0.

## Introduction

A smartphone has the ability to run an extensive range of applications under an operating system. Thus, it has expended the traditional purpose of a mobile phone, which is primarily used for phone-calling, message-texting, and photo-taking. As such, smartphones have become an integral part of our daily lives with a technical transformation that has subtly led to marked changes in our lifestyle and health habits. Children and adolescents, unsurprisingly, are the fastest-growing smartphone users, in terms of both ownership and usage. (Terras and Ramsay 2016) According to the 2019 Hong Kong census data, 81.3 percent of school-aged children between 10 and14 years old owned a smartphone, up from 46.1 percent in 2012. (Census and Statistics Department 2020) Similar trends were observed in the UK and the USA, where 91% (2020) (OfCom 2021) and 95% (2018) (Anderson and Jiang 2018), respectively, of teenagers reported smartphone ownership. School-aged children are also increasingly becoming heavy users of smartphones. The American Optometric Association (2017) found that children between the ages of 0 and 8 years old spent around 48 min a day on their mobile devices, compared to over 83% of school-aged children between 10 and 17 years old, who reported spending more than three hours per day on their digital devices. (American Optometric Association 2017) The 2020 Hong Kong census data showed similar trends with prolonged usage related to even longer internet usage times. Youth ages 10 years old and older spent around 30 h per week on the internet, which had more than doubled from 2001 reports of 12 h per week. (Census and Statistics Department 2020).

As defined by the American Optometric Association, the term “digital eye strain” (DES) refers to a set of vision and ocular disorders caused by extended use of digital devices. DES can affect anyone who spends a substantial amount of time focused on digital screens, whether for business or enjoyment, due to the rising usage of digital screens in everyday technology. Previous research has reported that the most common DES symptoms reported are dry eyes (Al Tawil et al. 2020) and its related symptoms, such as tearing, (Gammoh 2021) and symptoms related to accommodative stress, such as eyestrain, (Agarwal et al. 2013; Akinbinu 2013) headache, (Akinbinu 2013; Al Tawil et al. 2020) as well as neck or shoulder pain (Al Tawil et al. 2020). In addition, improper contact lens fit or comfort, improper refractive error correction, (Rosenfield et al. 2011) screen brightness, (Ahuja et al. 2021) screen position, (Agarwal et al. 2013; Coles-Brennan et al. 2019) poor ergonomic design of workstations, (Zayed et al. 2021) and low humidity environment (Zayed et al. 2021) can be risk factors for DES. However, most studies regarding DES have been conducted on working adult populations. It is only recently that concerns have been raised about whether children can cope with the visual demands of increased screen time. (Kozeis 2009) Young children and preteens are more vulnerable to DES because they have lower degree of self-control than adults. This is concerning, especially during developmental and puberty periods, where myopia progresses at a parallel pace. (Wong and Dahlmann-Noor 2020) The impact of prolonged smartphone use on a user’s eyesight is a major concern, particularly in Southeast Asia, where a disproportionately high percentage of people suffer from myopia. (Chua et al. 2015) It has previously been shown that there was a link between time spent on smartphones and refractive errors. With respect to screen time usage, it was found that the daily screen time, including smartphone, tablet, computers, and television, should be limited to two hours or less per day (Do et al. 2020) as it was associated with lower risk of refractive error progression. Only a few studies have quantified the impact of smartphone use alone on visual discomfort, (Choi et al. 2018; Yuan et al. 2021) as most DES studies combined smartphone use with that of other devices, such as tablets. Although previous studies (Demirayak et al. 2022; Mohan et al. 2021) reported that there was an increase in DES in children during the COVID-19 pandemic, the sample size was small (n = 692, age 9.72 ± 3.02 years; n = 217, age 13 ± 2.45 years). A recent systemic review (Wang et al. 2020) found no significant association of smartphone overuse with myopia, poor vision, or blurred vision from 10 cross-sectional studies. However, the results from four controlled trials revealed that the visual function scores of the smartphone overuse group were worse than the reduced-use group. To the best of our knowledge, no follow-up studies have investigated the effects of smartphone usage on DES. More importantly, studies among children and adolescents are limited, as earlier research focused on traditional digital screen viewers who were mainly computer office workers. (Rechichi et al. 2017) This study aims to investigate the prospective association between time spent on smartphones and DES among Hong Kong Chinese school-aged children.

## Material and methods

### Participants

Participants were recruited from 11 primary schools and 4 secondary schools in Hong Kong in 2017–2018. All students in the participating schools enrolled in P3-P5 and S1-S3 aged between 8 and 14 years were invited to participate. A total of 1,978 participants (response rate 60%) who agreed to participate were invited to attend a school health screening. All participants and their primary parental caregivers were also invited to complete a self-reported questionnaire at baseline and 1-year follow-up. A total of 1,508 students (80.7%) provided valid data in the DES questionnaire at baseline and were included in the present study. Of these, 1,298 (86%) completed the DES questionnaire at 1-year follow-up. Written consent was obtained from all participants at baseline. As all participants were under 18 years old, written parental consent was also obtained prior to their participation in this study. This study was approved by the Human Subjects Ethics Sub-committee of the Hong Kong Polytechnic University (HSEARS20151121001). All procedures of this study followed the guidelines of the Declaration of Helsinki.

## Data collection

### Self-administered questionnaire

The severity of DES was measured using a 10-item scale (Hayes et al. 2007) (double vision, blurred vision (reading), blurred vision (change from reading to distance viewing), difficulty in refocusing, eye strain, dry eyes, eye fatigue, irritated or burning eyes, photophobia, and headache). The participants rated each item with a score of 0 (none) to 10 (most severe). Given the highly skewed distribution of the scores, following the bimodal scoring method commonly used in symptom scales like the General Health Questionnaire-12 (Anjara et al. 2020), the item score was dichotomised into 0 if the response was 0 and 1 otherwise. The sum of the 10 dichotomised scores was used as the DES total score, and the Cronbach’s alpha of the baseline DES questionnaire was 0.86.

The time spent on smartphones per day was self-reported where participants reported the time spent on weekdays and weekends separately. The daily time spent was calculated by the weighted sum of the time spent on weekdays and weekends (that was, 5/7 × time spent on weekdays + 2/7 × time spent on weekends), and the time spent was categorised into five groups (0–60, 61–120, 121–180, 181–240, and 241 + min/d (Kwok et al. 2017)). Time spent on tablets per day was collected and processed in a similar manner. Time spent on moderate-to-vigorous physical activity was measured using the Global Physical Activity Questionnaire. (Bull et al. 2009).

Body Mass Index (BMI): Height (to the nearest 0.1 cm) and weight (to the nearest 0.1 kg) of the participants were measured by trained research assistants using SECA 213 portable stadiometer (SECA GMBH, Hamburg, Germany) and Tanita BMI body fat analyzer BC-541N (Tanita Health Equipment HK Ltd, Hong Kong). BMI was computed as weight (kg) / height (m)^2^.

### Caregiver questionnaire

The primary parental caregiver of each participant (decided among the caregivers) was invited to complete a self-administered questionnaire which collected basic demographic and socio-economic characteristics of the participants (type of accommodation, primary caregiver’s level of education, and monthly housing income).

## Data analysis

Univariate association between smartphone usage and DES was examined using one-way ANOVA, and a sex-stratified analysis was conducted to examine if there was any sex difference. Linear regressions were used to examine the association between smartphone usage and 1-year follow DES, controlling for possible confounders, including baseline DES, age, sex, BMI, time spent on tablets, time spent on moderate-to-vigorous physical activity, and caregiver-reported social-economic status. Two sets of analysis were performed, one with smartphone usage treated as a categorical variable (0–60, 61–120, 121–180, 181–240, and 241 + min/d) to test for any non-linear associations, and the second with smartphone usage treated as a continuous variable (h/d) to test for any linear associations. Missing confounders were imputed using multiple imputations. The full conditional specification method was used to impute the missing data and the averages of the results performed on 10 imputed datasets were reported. All data analysis was performed using SPSS 25.0. *P*-values of < 0.05 were considered significant and Bonferroni correction was made on multiple comparisons.

## Results

Table 1 shows the participants' characteristics. The sample was gender-balanced at baseline, where 639 males (85.4%) and 661 females (87.0%) remained at the 1-year follow-up. The mean age was 10.91 years (SD = 2.01). About half of the participants spent more than 4 h per day on smartphones, while less than 40% of them spent more than 1 h per day on tablets.

**Table 1:** Descriptive statistics (n = 1,508)

Variable	Mean	SD
Age (years)	10.9	2.0
BMI (kg/m^2^)	18.5	3.6
MVPA (hr/wk)	11.6	15.5
Variable	Frequency	Percentage
Sex		
Male	748	49.6
Female	760	50.4
Smartphone usage (min/day)		
0–60	277	18.4
61–120	171	11.3
121–180	134	8.9
181–240	160	10.6
241 +	766	50.8
Tablet usage (min/day)		
0–60	932	61.8
61–120	180	11.9
121–180	110	7.3
181–240	69	4.6
241 +	217	14.4
Parents' short sightedness		
Both	281	18.7
Only father	296	19.7
Only mother	225	15.0
Neither	505	33.7
Don't know	192	12.8
Type of accommodation		
Public housing	964	67.5
Home ownership scheme	182	12.7
Private housing	282	19.7
Primary caregiver's level of education		
No formal education	16	1.1
Primary	170	12.1
Secondary	1,067	76.0
Tertiary or above	151	10.8
Monthly household income (Hong Kong dollar)		
0–9,999	207	14.9
10,000–19,999	553	39.9
20,000–29,999	329	23.7
30,000–39,999	154	11.1
40,000–49,999	70	5.1
50,000 +	73	5.3

BMI: Body Mass Index, MVPA: moderate-to-vigorous physical activity (MVPA)

Tables 2 and 3 show the frequencies of reporting the 10 DES symptoms and the total DES score by smartphone usage. The most commonly reported symptoms were eye fatigue (n = 804, 53.3%), blurred vision when changing from reading to distance viewing (n = 586, 38.9%), and irritated or burning eyes (n = 516, 34.2%). The average DES total score at baseline was 2.91 (SD = 2.90), which increased to 3.20 (SD = 3.19) at the 1-year follow-up. One-way ANOVA results show that smartphone usage was positively associated with all DES symptoms at baseline and 1-year follow-up and total DES score at baseline (all *P*s < 0.05). Stratified analysis shows the same pattern across sex (Supplementary Tables S1–S4).

**Table 2:** Baseline smartphone usage (h/d) and baseline digital eye strain (n = 1,508)

Smartphone usage (min/d)	Double vision	Blurred vision (reading)	Blurred vision (change from reading to distance viewing)	Difficulty in refocusing	Eye strain	Dry eyes	Eye fatigue	Irritated or burning eyes	Photophobia	Headache	Total score (SD)
0–60 (n = 277)	18.1%	23.8%	27.8%	21.7%	14.4%	6.5%	42.6%	26.4%	9.8%	23.8%	2.15 (2.50)
61–120 (n = 171)	16.4%	22.8%	30.4%	21.6%	15.8%	11.1%	48.5%	25.7%	9.9%	26.3%	2.29 (2.58)
121–180 (n = 134)	21.6%	29.9%	31.3%	23.9%	17.2%	7.5%	46.3%	24.6%	10.5%	32.8%	2.46 (2.60)
181–240 (n = 160)	21.9%	30.0%	37.5%	21.3%	20.0%	11.3%	50.0%	28.1%	16.3%	32.5%	2.69 (2.63)
241 + (n = 766)	24.5%	38.6%	46.3%	35.3%	23.4%	15.4%	60.2%	41.9%	21.4%	38.1%	3.45 (3.09)
p-value	0.07	< 0.001	< 0.001	< 0.001	0.01	0.001	< 0.001	< 0.001	< 0.001	< 0.001	< 0.001
p-value for trend	0.006	< 0.001	< 0.001	< 0.001	< 0.001	< 0.001	< 0.001	< 0.001	< 0.001	< 0.001	< 0.001

**Table 3:** Baseline smartphone usage (h/d) and 1-year follow-up digital eye strain (n = 1,298)

Smartphone usage (min/d)	Double vision	Blurred vision (reading)	Blurred vision (change from reading to distance viewing)	Difficulty in refocusing	Eye strain	Dry eyes	Eye fatigue	Irritated or burning eyes	Photophobia	Headache	Total score (SD)	Change from baseline to 1-year follow-up (SD)
0–60 (n = 225)	14.9%	24.9%	34.4%	24.8%	14.8%	10.5%	47.0%	26.8%	12.2%	24.4%	2.35 (2.63)	0.23 (2.84)
61–120 (n = 142)	15.1%	31.5%	37.0%	21.9%	23.1%	17.1%	51.0%	32.9%	14.3%	31.3%	2.76 (3.09)	0.47 (3.23)
121–180 (n = 117)	23.6%	35.0%	45.5%	36.6%	17.1%	11.7%	45.5%	34.4%	14.9%	33.3%	2.99 (3.07)	0.69 (3.14)
181–240 (n = 146)	26.0%	37.7%	49.3%	34.3%	24.7%	18.5%	51.4%	37.7%	24.0%	32.9%	3.36 (3.19)	0.73 (2.91)
241 + (n = 670)	22.0%	38.4%	48.1%	37.6%	25.4%	18.1%	58.8%	44.4%	24.6%	40.1%	3.58 (3.34)	0.11 (3.44)
p-value	0.02	0.005	0.001	< 0.001	0.008	0.043	0.004	< 0.001	< 0.001	< 0.001	< 0.001	0.14
p-value for trend	0.008	< 0.001	< 0.001	< 0.001	0.001	0.01	< 0.001	< 0.001	< 0.001	< 0.001	< 0.001	0.33

The adjusted means of the total DES scores are reported in Table 4. The full adjustment analysis (Model 2) with Bonferroni adjustment (level of significance = 0.05/10 = 0.005) shows that participants with baseline smartphone usage of 241 + min/d had a significantly higher baseline total DES score than those with baseline smartphone usage of 0–60 min/d (2.44 vs 3.21, *P* = 0.00002). Participants with baseline smartphone usage of 181—240 min/d had a significantly higher 1-year follow-up total DES score than those with baseline smartphone usage of 0–60 h/d (2.80 vs 3.50, *P* = 0.003). An hour increase per day on baseline smartphone usage was associated with a 0.09-point increase in baseline DES (*P* < 0.001). The association with DES at 1-year follow-up was however, not significant (*P* = 0.43). When adjusted for confounding factors, such as demographics and socio-economic status, the positive association between duration of smartphone usage and DES remained significant (all *P*s < 0.001 except for double vision).

**Table 4:** Estimated marginal means (95% CI) of digital eye strain score

		Baseline digital eye strain score (n = 1,508)	
Baseline smartphone usage (min/d)	Crude	Model 1	Model 2
0–60	2.15 (1.68, 2.61)	2.46 (2.13, 2.80)	2.44 (2.02, 2.87)
61–120	2.29 (1.87, 2.71)	2.55 (2.10, 3.00)	2.56 (2.11, 3.02)
121–180	2.46 (1.94, 2.97)	2.61 (2.14, 3.08)	2.59 (2.12, 3.07)
181–240	2.69 (2.35, 3.02)	2.71 (2.39, 3.03)	2.70 (2.17, 3.24)
241 +	3.45^a,b,c,d^ (3.11, 3.80)	3.24^a,b^ (3.03, 3.46)	3.21^a^ (2.88, 3.53)
Baseline smartphone usage as a continuous variable (h/d)	0.15*** (0.11, 0.18)	0.10*** (0.06, 0.14)	0.09*** (0.06, 0.13)
		1-year follow-up digital eye strain score (n = 1,298)	
Baseline smartphone usage (min/d)	Crude	Model 1	Model 2
0–60	2.60 (2.21, 3.00)	2.85 (2.52, 3.19)	2.80 (2.40, 3.20)
61–120	2.92 (2.47, 3.37)	3.13 (2.63, 3.62)	3.08 (2.61, 3.55)
121–180	3.23 (2.60, 3.86)	3.36 (2.83, 3.90)	3.29 (2.60, 3.98)
181–240	3.52^a^ (3.03, 4.01)	3.55^a^ (3.13, 3.97)	3.50^c^ (3.03, 3.98)
241 +	3.39^a^ (3.17, 3.60)	3.22 (3.10, 3.34)	3.19 (2.85, 3.53)
Baseline smartphone usage as a continuous variable (h/d)	0.06* (0.01, 0.11)	0.02 (-0.04, 0.07)	0.02 (-0.03, 0.07)

Model 1 adjusted for age and sex

Model 2 adjusted for age, sex, baseline digital eye strain score, parents' short sightedness, BMI, time spent on moderate-to-vigorous physical activity, and caregiver-reported socio-economic status

^*^ / ** / *** significant at 5% / 1% / 0.1% level

^a^significant different (p < 0.005, Bonferroni adjustment) with baseline smartphone usage of 0–60 min/d

^b^significant different (p < 0.005, Bonferroni adjustment) with baseline smartphone usage of 61–120 min/d

^c^significant different (p < 0.005, Bonferroni adjustment) with baseline smartphone usage of 121–180 min/d

^d^significant different (p < 0.005, Bonferroni adjustment) with baseline smartphone usage of 181–240 min/d

## Discussion

To the best of our knowledge, this is the first study examining smartphone usage across a one-year time span and assessing the association between baseline smartphone usage pattern and self-reported DES in school-aged children. Many studies have shown that uncomfortable sensations, including irritation, burning, and redness, as well as eyestrain, blurry and double vision, and fatigue are linked to DES.(Rosenfield 2011) Adult video display terminal users have endured multiple DES symptoms over the past three decades.(Sheedy 1992) Their prevalence has markedly increased due to the influence of the nearly universal exposure of digital screens. Interestingly, our school-aged population group, particularly those with greater smartphone usage time (> 4 h/d) reportedly experienced quite a high incidence of associated digital eye discomfort symptoms similar to those reported by our adult video display terminal predecessors. (Rosenfield 2011) These included eye fatigue experienced by 60.2%, followed by blurred distance vision after studying or other related near work (46.3%), irritated or burning eyes (41.9%), and headaches (38.1%). The increased incidence of DES, in accordance with time spent on a smartphone at baseline and after a year, is also consistent with cross-sectional reports on digital device usage in children and adolescents. (Das et al. 2016; Rechichi et al. 2017) While the latter study (Rechichi et al. 2017) was related to video game play screen time, a previous study conducted in Hong Kong revealed more frequent smartphone usage, mainly on instant messaging, which doubled their reported time engaged in mobile games. (Lee et al. 2021) In most cases, it was noted that their usage profile involved a different pattern compared to other studies, which only explored video gaming habits. (Gentile 2009) Here, the self-reported time spent on instant messaging involved shorter screen time, but more frequent sending and receiving of instant text messages. In comparison, video game playing involved longer hours of duration per session. Despite these two very distinct digital screen usage patterns, similar visual problems emerged as the total time spent on digital screens increased. There are already published reports on various warning signs of increased digital screen time. These include early adoption and the growing use of mobile devices, even amongst low-income and minority communities. Similarly, more than 54% of our school-aged children represented participants from below-average income households. As such, the data implied that they were no less susceptible to the effects of increased digital screen time usage. This growth in the number of smartphone users is likely to translate to prolonged usage among the younger generations of these early adopters, (Kabali et al. 2015) in whom possession at a young age may easily lead to smartphone addiction. For example, the health department guidelines from Australia suggested that 5- to 17-year-old youths should limit recreational screen time to no more than 2 h per day. (https://www.health.gov.au/health-topics/physical-activity-and-exercise/physical-activity-and-exercise-guidelines-for-all-australians) However, 70.3% of our participants reported spending more than 2 h per day on their smartphones alone, which already exceeded the guidelines for their age group. It has been reported that students whose first age of smartphone ownership is 13 and below have a higher level of smartphone addiction than those whose age of first smartphone is 16 and above. (Sahin et al. 2013) There is a direct link between smartphone use and smartphone dependence. (Borkotoky and Saikia 2019) The follow-up data of the current study also supported a preliminary pattern, in which most school-aged children reported an increase in hours of usage at the one-year follow-up.

According to World Bank data, children under 15 years old make up 25% of the world population.(The World Bank 2019) The concerning symptoms, such as eye fatigue and symptoms associated with increased smartphone usage, were reported by participants as young as 8 years old. These presenting symptoms may be indicators of potentially undiagnosed accommodative and vergence dysfunctions, which require further optometric workup and management. It will be interesting to see if these symptoms can be alleviated by proper refractive correction, (Loh and Redd 2008) vision therapy intervention, or by lifestyle/screen time modification and ergonomics positioning in the future. Numerous studies have reported that improper vision correction and/or disorders do not only create a public health problem, but also have a tremendous impact on academic and sports performance, as well as future employment opportunities. The results of a previous study by members of this team also suggested that viewing electronic displays for longer than 2 h per day was significantly related to refractive error progression. (Do et al. 2020).

Several studies have consistently shown that myopic students spend significantly more time (about 32 to 42 min/day) on near-work activities, including computer and smartphone usage, than non-myopic students. (Moon et al. 2016) Significantly severe DES symptoms have been reported in individuals with uncorrected refractive errors as low as 0.50 D (either sphere or cylinder). (Daum et al. 1988; Wiggins and Daum 1991) Thus, it is important not to overlook DES with smartphone usage habits as a potentially contributing factor to related chief complaints, even in the younger population group.

This study has several limitations. First, the data was collected through a self-reported questionnaire which was commonly used in epidemiological studies (Hayes et al. 2007). No thorough optometric examination was carried out to evaluate if reported symptoms correlated with clinical DES-related dysfunctions in accommodative, oculomotor, and ocular surface health status. Respondents may have potentially under-or over-estimated their symptoms even though care has been taken with available staff onsite to assist during questionnaire intake. Similarly, time spent on smartphone was also self-reported and its validity was doubtful (Lee et al. 2021). However, recruitment of a large sample size to increase the statistical power and internal validity of our results, which have shown that DES significantly worsened from baseline to one-year follow-up, suggests these aforementioned limitations were minimal. Second, concurring visual problems that can worsen DES symptoms such as uncorrected/under-corrected refractive errors, contact lens overwear, adverse effects from common medications that can affect accommodative/oculomotor functions should be reviewed and examined in an optometry clinic in future studies. Optimizing awareness and providing guidance regarding ophthalmic conditions that should not be left untreated in the school-aged population remains an ever-growing challenge. With increasing digital device usage around our environment, the focus on DES may have enhanced a collective concern among parents, schools, and public health units to explore modifiable and correctable variables that could better improve visual hygiene and reduce the volume of DES reported.

This study, along with many others, has consistently demonstrated that DES has a cumulative effect that cannot be ignored, even though symptoms may be transient. (Khalaj et al. 2015) These preliminary data reinforce the importance of substantiating a longer and objective follow-up of the visual effects smartphone use may pose on school-aged population users. The current data related to this topic is scarce but important, as this age group is still undergoing vulnerability in visual development. Early diagnosis and prevention are the most effective strategies for dealing with DES. Modifications to the ergonomics of the working environment, patient education, and proper eye care are critical in the management of DES. It is unwise to wait until children exhibit symptoms such as eye fatigue, blurred vision, or irritated or burring eyes, before restricting smartphone usage time. It is critical to teach children how to use this tool safely and raise public awareness about good eye care practices.

## Supplementary Information

Below is the link to the electronic supplementary material.

Supplementary file1 (DOCX 37 KB)

## Data Availability

The data used in the current study are available upon reasonable request.

## Abbreviations

DES: Digital Eye Strain
GPAQ: Global Physical Activity Questionnaire
MVPA: Moderate-to-vigorous Physical Activity
BMI: Body Mass Index
VDT: Video Display Terminal
