# Supplementary material for: Association between time spent on smartphones and digital eye strain: A 1-year prospective observational study among Hong Kong children and adolescents
Source: Environ Sci Pollut Res Int. 2023 Mar 29;30(20):58428–35. doi: 10.1007/s11356-023-26258-0 (PMC10057686; doi:10.1007/s11356-023-26258-0)
Supplement: Supplementary file 1 — Supplementary file1 (DOCX 37 KB) [file 11356_2023_26258_MOESM1_ESM.docx]

Table S1. Baseline smartphone usage (h/d) and baseline digital eye strain (boys, n=748)

|  | Double vision | Blurred vision (reading) | Blurred vision (change from reading to distance viewing) | Difficulty in refocusing | Eye strain | Dry eyes | Eye fatigue | Irritated or burning eyes | Photophobia | Headache | Total score (SD) |
| --- | --- | --- | --- | --- | --- | --- | --- | --- | --- | --- | --- |
| Smartphone usage (h/d) |  |  |  |  |  |  |  |  |  |  |  |
| 0-1 (n=135) | 19.3% | 22.2% | 27.4% | 22.2% | 17.8% | 5.9% | 41.5% | 28.9% | 11.9% | 23.7% | 2.21 (2.54) |
| 1-2 (n=73) | 16.4% | 26.0% | 34.2% | 21.9% | 13.7% | 12.3% | 38.4% | 28.8% | 11.0% | 28.8% | 2.32 (2.74) |
| 2-3 (n=82) | 22.0% | 30.5% | 29.3% | 23.2% | 19.5% | 8.5% | 43.9% | 22.0% | 11.0% | 29.3% | 2.39 (2.56) |
| 3-4 (n=80) | 23.8% | 35.0% | 38.8% | 23.8% | 23.8% | 13.8% | 47.5% | 25.0% | 15.0% | 27.5% | 2.74 (2.85) |
| 4+ (n=378) | 24.9% | 37.0% | 44.7% | 33.6% | 19.8% | 13.5% | 56.6% | 40.0% | 22.2% | 35.2% | 3.28 (3.05) |
| p-value | 0.46 | 0.02 | 0.02 | 0.03 | 0.60 | 0.15 | 0.003 | 0.002 | 0.008 | 0.13 | 0.001 |
| p-value for trend | 0.08 | 0.001 | <0.001 | 0.003 | 0.35 | 0.02 | <0.001 | 0.004 | 0.001 | 0.01 | <0.001 |

Table S2. Baseline smartphone usage (h/d) and 1-year follow-up digital eye strain (boys, n=664)

|  | Double vision | Blurred vision (reading) | Blurred vision (change from reading to distance viewing) | Difficulty in refocusing | Eye strain | Dry eyes | Eye fatigue | Irritated or burning eyes | Photophobia | Headache | Total score (SD) | Change from baseline to 1-year follow-up (SD) |
| --- | --- | --- | --- | --- | --- | --- | --- | --- | --- | --- | --- | --- |
| Smartphone usage (h/d) |  |  |  |  |  |  |  |  |  |  |  |  |
| 0-1 (n=110) | 13.6% | 23.6% | 32.4% | 24.3% | 14.4% | 11.7% | 41.4% | 28.4% | 15.3% | 24.3% | 2.35 (2.89) | 0.18 (2.99) |
| 1-2 (n=62) | 19.4% | 34.4% | 32.8% | 21.3% | 22.6% | 14.8% | 46.8% | 29.5% | 17.7% | 33.9% | 2.76 (3.28) | 0.38 (3.87) |
| 2-3 (n=74) | 28.4% | 33.8% | 41.9% | 37.8% | 16.2% | 15.1% | 35.6% | 32.9% | 17.8% | 32.4% | 2.97 (3.35) | 0.63 (3.33) |
| 3-4 (n=73) | 31.5% | 39.7% | 52.1% | 34.3% | 24.7% | 23.3% | 50.7% | 45.2% | 30.1% | 31.5% | 3.63 (3.51) | 0.93 (2.56) |
| 4+ (n=345) | 22.6% | 36.4% | 44.5% | 36.4% | 24.1% | 17.9% | 52.2% | 40.8% | 24.1% | 37.3% | 3.36 (3.36) | 0.09 (3.77) |
| p-value | 0.04 | 0.13 | 0.04 | 0.04 | 0.18 | 0.30 | 0.06 | 0.048 | 0.09 | 0.16 | 0.04 | 0.35 |
| p-value for trend | 0.08 | 0.03 | 0.009 | 0.006 | 0.04 | 0.13 | 0.02 | 0.006 | 0.02 | 0.02 | 0.003 | 0.63 |

Table S3. Baseline smartphone usage (h/d) and baseline digital eye strain (girls, n=760)

|  | Double vision | Blurred vision (reading) | Blurred vision (change from reading to distance viewing) | Difficulty in refocusing | Eye strain | Dry eyes | Eye fatigue | Irritated or burning eyes | Photophobia | Headache | Total score (SD) |
| --- | --- | --- | --- | --- | --- | --- | --- | --- | --- | --- | --- |
| Smartphone usage (h/d) |  |  |  |  |  |  |  |  |  |  |  |
| 0-1 (n=142) | 16.9% | 25.4% | 28.2% | 21.1% | 11.3% | 7.0% | 43.7% | 23.9% | 7.8% | 23.9% | 2.09 (2.47) |
| 1-2 (n=98) | 16.3% | 20.4% | 27.6% | 21.4% | 17.4% | 10.2% | 56.1% | 23.5% | 9.2% | 24.5% | 2.27 (2.47) |
| 2-3 (n=52) | 21.2% | 28.9% | 34.6% | 25.0% | 13.5% | 5.8% | 50.0% | 28.9% | 9.6% | 38.5% | 2.56 (2.69) |
| 3-4 (n=80) | 20.0% | 25.0% | 36.3% | 18.8% | 16.3% | 8.8% | 52.5% | 31.3% | 17.5% | 37.5% | 2.64 (2.41) |
| 4+ (n=388) | 24.2% | 40.2% | 47.9% | 36.9% | 26.8% | 17.3% | 63.7% | 43.8% | 20.6% | 41.0% | 3.62 (3.12) |
| p-value | 0.27 | <0.001 | <0.001 | <0.001 | 0.001 | 0.004 | 0.001 | <0.001 | 0.001 | 0.001 | <0.001 |
| p-value for trend | 0.03 | <0.001 | <0.001 | <0.001 | <0.001 | 0.001 | <0.001 | <0.001 | <0.001 | <0.001 | <0.001 |

Table S4. Baseline smartphone usage (h/d) and 1-year follow-up digital eye strain (girls, n=685)

|  | Double vision | Blurred vision (reading) | Blurred vision (change from reading to distance viewing) | Difficulty in refocusing | Eye strain | Dry eyes | Eye fatigue | Irritated or burning eyes | Photophobia | Headache | Total score (SD) | Change from baseline to 1-year follow-up (SD) |
| --- | --- | --- | --- | --- | --- | --- | --- | --- | --- | --- | --- | --- |
| Smartphone usage (h/d) |  |  |  |  |  |  |  |  |  |  |  |  |
| 0-1 (n=119) | 16.0% | 26.1% | 36.1% | 25.2% | 15.1% | 14.4% | 52.1% | 25.2% | 9.2% | 24.4% | 2.36 (2.37) | 0.28 (2.71) |
| 1-2 (n=84) | 11.9% | 29.4% | 40.0% | 22.4% | 23.5% | 22.6% | 54.1% | 35.3% | 11.8% | 29.4% | 2.76 (2.97) | 0.54 (2.73) |
| 2-3 (n=49) | 16.3% | 36.7% | 51.0% | 34.7% | 18.4% | 16.2% | 60.4% | 36.7% | 10.4% | 34.7% | 3.02 (2.60) | 0.80 (2.86) |
| 3-4 (n=73) | 20.6% | 35.6% | 46.6% | 34.3% | 24.7% | 24.7% | 52.1% | 30.1% | 17.8% | 34.3% | 3.10 (2.83) | 0.52 (3.22) |
| 4+ (n=360) | 21.4% | 40.3% | 51.7% | 38.7% | 36.8% | 24.1% | 65.2% | 47.9% | 25.1% | 42.9% | 3.79 (3.31) | 0.13 (3.10) |
| p-value | 0.27 | 0.048 | 0.03 | 0.01 | 0.11 | 0.0502 | 0.04 | <0.001 | <0.001 | 0.003 | <0.001 | 0.52 |
| p-value for trend | 0.049 | 0.002 | 0.002 | 0.001 | 0.01 | 0.04 | 0.006 | <0.001 | <0.001 | <0.001 | <0.001 | 0.37 |
